# Nitric oxide impacts on angiotensin AT2 receptor modulation of high-pressure baroreflex control of renal sympathetic nerve activity in anaesthetized rats

**DOI:** 10.1111/apha.12207

**Published:** 2013-12-17

**Authors:** M H Abdulla, E J Johns

**Affiliations:** Department of Physiology, Western Gateway Building, University College CorkCork, Ireland

**Keywords:** AT2 receptors, high-pressure baroreflex, nitric oxide

## Abstract

**Aim:**

Nitric oxide (NO) interacts with the local brain renin-angiotensin system to modulate sympathetic outflow and cardiovascular homoeostasis. This study investigated whether NO influenced the ability of angiotensin AT2 receptor activation to modify the high-pressure baroreceptor regulation of renal sympathetic nerve activity (RSNA) and heart rate (HR).

**Methods:**

Anaesthetized (chloralose/urethane) rats were prepared to allow generation of baroreflex gain curves for RSNA or HR following intracerebroventricular (I.C.V.) CGP42112 (AT2 receptor agonist), PD123319 (AT2 receptor antagonist) or losartan (AT1 receptor antagonist), and then in combination with L-NAME (NO synthase inhibitor).

**Results:**

I.C.V. PD123319, CGP42112, and Losartan did not change baseline mean arterial pressure, HR or RSNA. Baroreflex sensitivities for RSNA and HR were increased following AT2 receptor activation with CGP42112 by 112 and 157%, respectively, but were reduced following PD123319 by 20% (all *P* < 0.05). L-NAME alone increased baroreflex sensitivity for both RSNA and HR, by 62 and 158%, respectively, but when co-infused with either CGP42112 or PD123319, the baroreflex sensitivity fell to values comparable to those obtained during I.C.V. saline infusion. The baroreflex sensitivities for RSNA and HR were increased by losartan by 92% and 192%, respectively, but in the presence of L-NAME were no different from those obtained during I.C.V. saline infusion.

**Conclusion:**

There is an important facilitatory role for AT2 receptors in the high-pressure baroreflex regulation of RSNA and HR which is dependent on a functional NO/NOS system. Conversely, AT1 receptors have an inhibitory effect on the baroreflex, an action that relies on a tonic inhibition of NO.

Nitric oxide (NO) generated within the central nervous system may act as a neurotransmitter or neuromodulator of baroreflex regulation of blood pressure (Qadri *et al*. 1999, Patel *et al*. 2001). Injection of NO into specific brain regions such as the paraventricular nucleus (PVN) led to a sympathetically mediated decrease in blood pressure (Horn *et al*. 1994), while systemic blockade of neuronal nitric oxide synthase enzyme (nNOS) was found to reduce the lower plateau of the heart rate baroreflex curve without affecting the renal sympathetic nerve activity (RSNA) baroreflex control in conscious rabbits (Murakami *et al*. 1998).

The colocalization of the NOS enzyme and AT1 receptor mRNA in several brain regions in rats indicates a possible interaction between central NO and angiotensin II (Ang II) at the level of AT1 receptors (Krizanova *et al*. 2001). This relationship could go part way to explaining the inhibitory action of NO on sympathetic out flow when exogenous Ang II was infused intravenously in conscious rabbits (Liu *et al*. 1998). Immunolabeling studies have revealed that NO production in somata and dendrites of nucleus tractus solitarius (NTS) neurones is enhanced by AT1 receptor antagonism, but, significantly, this effect was prevented when AT2 receptors on these neurones were blocked (Wang *et al*. 2012). Moreover, it has been suggested that the vasodepressor effect of AT2 receptor after footshock challenges in rats is dependent on NO release in the brain as well as peripherally (Sosa-Canache *et al*. 2000).

Although the impact of endogenous NO on AT1 receptor-mediated control of RSNA has been explored (Kumagai *et al*. 1993, Eshima *et al*. 2000), there is less known regarding the contribution of centrally generated NO on AT2 receptor activation in the baroreflex regulatory mechanism in rats. *In vitro* studies have revealed that NO synthase enzyme (NOS) blockade using *N*^*ω*^-nitro-L-arginine methyl ester (L-NAME) inhibited the facilitatory role of AT2 receptors on neuronal membrane potassium currents (Kang *et al*. 1993, Gao & Zucker 2010). However, a recent *in vivo* study from this laboratory showed significant yet independent roles for AT2 receptors and NO in the sympatho-inhibitory reflex mechanism initiated by acute saline volume expansion (Abdulla & Johns 2013).

The present investigation tested the hypothesis that NO produced within the central nervous system contributed to the impact of AT2 receptor activation on the ability of the arterial baroreceptors to reflexly regulate heart rate and renal sympathetic nerve activity. To this end, the effect of central administration of CGP42112, a selective AT2 receptor agonist, or PD123319, a selective AT2 receptor antagonist on high-pressure baroreceptor function, was compared in the presence or absence of the NOS inhibitor L-NAME.

## Methods

Experiments were conducted using 275–350 g body weight male Wistar rats purchased from Harlan (Harlan, UK) and maintained in the Biological Service Unit at University College Cork, Cork, Republic of Ireland. The rats were fed a standard rat chow and tap water *ad libitum* and were under a 12:12-h dark–light regime at 20 ± 3 °C and 35% humidity. All procedures on rats were in agreement with national guidelines and the European Community Directive 86/609/EC and with the endorsement of the local Animal Experimentation Ethical Committee at University College Cork.

### Surgical procedure

Rats were fasted overnight and anaesthetized with an intraperitoneal injection of a mixture of chloralose and urethane (Sigma-Aldrich Company, Gillingham, Dorset, UK) (16.5 and 250 mg mL− respectively) of 1–1.2 mL initially with supplementation of 0.05 mL of the same anaesthetic I.V. given every 30 min. The trachea was cannulated to allow free air passage. The right femoral artery was cannulated (PE 50, Portex, Kent, UK), and the cannula was connected to a fluid-filled pressure transducer attached to a quad bridge amplifier (ADInstruments, Hastings, UK) for continuous mean arterial pressure (MAP) and heart rate (HR) measurement, while the femoral vein was catheterized for infusion of saline (150 mm NaCl, 3 mL h−), drugs and supplementary anaesthetic. For acute implantation of the I.C.V. cannula, the rat head was positioned in a stereotaxic frame (Kopf Instruments, Tujunga, CA, USA) and a midline incision was made through the skin. A small hole was drilled into the skull, and a stainless steel cannula with diameter of 0.82 mm was inserted into the site 1.0 mm posterior to the bregma, 2.5 mm lateral to the midline and 2.55 mm ventral to the surface of the dura as previously described (Huang & Johns 1998, Houghton *et al*. 2010). Correct placement of the cannula tip intraventricularly was established by slow extrusion of cerebrospinal fluid from the end of the cannula (Wainford & Kapusta 2009) or by Evans blue dye injection at the end of the study to check the distribution of dye through the ventricles (Huang & Johns 1998). The I.C.V. cannula was connected to a PE 50 tube (Portex) connected to a 25 *μ*L microsyringe (Hamilton, USA) fitted to a microinfusion pump (KD Scientific; Linton Instruments, Norfolk, UK). The left kidney was exposed retroperitoneally, and a renal sympathetic nerve bundle running on or beside the renal artery was separated from the surrounding tissues, with the use of a dissecting microscope, and sealed in place onto multi-stranded stainless steel wire electrodes using a two-component silicon glue (Klasse4Dental, Augsburg, Germany).

A high impedance head stage attached to a low noise/high gain amplifier (NeuroAmp EX®, ADInstruments) was used to record RSNA. The raw signals from the amplifier were distributed between an audio amplifier, to enable auditory evaluation of the signal and a PowerLab data acquisition system connected to a computer where the raw and integrated signals were displayed. RSNA was amplified and filtered (gain 100x; high-and low-pass filters set at 100–2 KHz respectively), digitized at 1000 Hz s^−1^ and stored for later analysis. LabChart 7 software (ADInstruments) was used to process and analyse the data. The integrated signal was utilized to study the baroreceptor regulation of renal sympathetic out flow. Raw signals of pulsatile blood pressure were used to generate MAP and HR.

### Protocols

A stabilization period of at least 2 h after the surgical procedure was allowed. I.C.V. administration of saline or drugs was initiated for 10 min at 30 *μ*L h^−1^ followed by a maintenance infusion of 7.5 *μ*L h^−1^ throughout the experiment. Baseline MAP, HR and RSNA were recorded for 5 min after which the first baroreflex curves for RSNA and HR were generated. This was performed using a slow ramp increase and decrease in arterial blood pressure by 50–60 mmHg, following an I.V. infusion of phenylephrine (PE) or sodium nitroprusside (SNP), respectively, at a dose of 10 *μ*g in a volume of 0.2 mL of saline for each which was infused at a rate of 18 mL h^−1^ over 40 s. A time period of 10–15 min was allowed following the infusion of phenylephrine and sodium nitroprusside for all the variables to return to baseline. Following the first baroreflex curve generation, a recovery period of at least 2 h was allowed before the second I.C.V. drug administration and baroreflex curve generation for RSNA and HR was repeated in a similar fashion. The I.C.V. maintenance infusion of saline or drug was continued throughout the 30 min baroreflex curve generation period, and haemodynamic and RSNA data were continuously collected throughout the study. A 5-min recording of baseline MAP, HR and RSNA was taken before and after the first and second I.C.V. infusion. The rats were killed at the end of the experimental protocol using an overdose of anaesthetic, and 30 min later, the background noise was recorded. The background noise value was subtracted from all original RSNA recordings and used during data analysis.

### Experimental groups

Nine groups of rats were used

*Group 1* (Saline/saline) (*n* = 5): This group served as a time control which received saline in the first I.C.V. infusion throughout the baroreflex curve generation. The second I.C.V. saline infusion was administered in a similar pattern to the first one.
*Group 2* (Saline/L-NAME) (*n* = 7): In this group, saline was infused I.C.V. throughout the first baroreflex curve generation. The I.C.V infusion was then changed to nitro-L-arginine methyl ester, an NO synthase enzyme inhibitor (L-NAME, 150 *μ*g kg^−1^ min^−1^) at 30 *μ*L h^−1^ for 10 min loading followed by 7.5 *μ*L h^−1^ to deliver one-quarter of the dose as maintenance throughout the second baroreflex curve generation (Abdulla & Johns 2013). This dosage level was comparable to that used previously and reported to non-selectively inhibit brain NOS (Moore *et al*. 1991, Kadekaro *et al*. 1998, Dobrucki *et al*. 2001, Raimondi *et al*. 2007).
*Group 3* (Saline/CGP) (*n* = 6): This group received a saline I.C.V. infusion throughout the first baroreflex curve generation. In the second I.C.V. infusion, a selective AT2 receptor agonist CGP42112 (CGP, 50 *μ*g kg^−1^ min^−1^) was given at 30 *μ*L h^−1^ for 10 min loading followed by a 7.5 *μ*L h^−1^ to deliver one-quarter of the dose as maintenance (Gao *et al*. 2008, Abdulla & Johns 2013) throughout the second baroreflex curve generation.
*Group 4* (Saline/PD) (*n* = 7): Similar to the previous groups, saline in this group was infused in the first I.C.V. infusion throughout the baroreflex curve generation. Thereafter, the I.C.V. infusion was changed to a selective AT2 receptor antagonist, PD123319 (de Gasparo *et al*. 1995) (PD, 50 *μ*g kg^−1^ min^−1^) delivered at 30 *μ*L h^−1^ for 10 min loading followed by 7.5 *μ*L h^−1^ to deliver one-quarter of the dose as maintenance (Gao *et al*. 2008, Abdulla & Johns 2013) throughout the second baroreflex curve generation.
*Group 5* (Saline/losartan) (*n* = 7): Saline was infused I.C.V during the first baroreflex curve generation after which the I.C.V. infusion was changed to one containing a selective AT1 receptor antagonist (7.5 *μ*g kg^−1^ min^−1^), losartan, that was delivered at 30 *μ*L h^−1^ for 10 min loading followed by 7.5 *μ*L h^−1^ maintenance dose (Huang & Johns 2001, Abdulla & Johns 2013) throughout the second baroreflex curve generation.
*Group 6* (PD/PD+L-NAME) (*n* = 7): This group received I.C.V. an infusion of PD (50 *μ*g kg^−1^ min^−1^) throughout the first baroreflex curve generation. The I.C.V. infusion was then switched to PD plus L-NAME (50 + 150 *μ*g kg^−1^ min^−1^ respectively) at 30 *μ*L h^−1^ for 10 min loading followed by 7.5 *μ*L h^−1^ to deliver one-quarter of the dose as maintenance throughout the second baroreflex curve generation (Abdulla & Johns 2013).
*Group 7* (L-NAME/L-NAME+PD) (*n* = 7): L-NAME (150 *μ*g kg^−1^ min^−1^) was infused I.C.V. for the generation of the first baroreflex curve, and L-NAME and PD (150 + 50 *μ*g kg^−1^ min^−1^) were delivered I.C.V. during the generation of the second baroreflex curve.
*Group 8* (CGP/CGP+L-NAME) (*n* = 7): CGP (50 *μ*g kg^−1^ min^−1^) at 30 *μ*L h^−1^ was infused throughout the first baroreflex curve generation, and then, the I.C.V. infusion was switched to CGP plus L-NAME (50 + 150 *μ*g kg^−1^ min^−1^) for the second baroreflex curve generation.
*Group 9* (Losartan/losartan+L-NAME) (*n* = 7): During the generation of the baroreflex curves in this group, losartan (7.5 *μ*g kg^−1^ min^−1^) was infused as in the first I.C.V. infusion and then losartan plus L-NAME (7.5 + 150 *μ*g kg^−1^ min^−1^) for the second infusion before the production of the baroreflex curves.

## Materials

The chemicals used included the following: phenylephrine hydrochloride, sodium nitroferricyanide (III) dehydrate (sodium nitroprusside), *N*^*ω*^-nitro-L-arginine methyl ester (L-NAME), CGP42112, PD123319, losartan potassium, chloralose and urethane (Sigma-Aldrich Company).

## Data analysis

The averaged background noise (30 min after killing the animal) for integrated RSNA was calculated and subtracted from all RSNA recordings prior to any analysis. The baseline values of MAP, HR and integrated RSNA were recorded for 5 min immediately prior to the start of the I.C.V. injections and then over the final 5 min of the infusion were presented as the actual values and were compared using a paired Student's *t*-test. The analysis of the baroreflex curves for the relationship between MAP and integrated RSNA or HR was performed by fitting data to a logistic sigmoid function [*y *= *A*1/(1 + *exp*(*A*2(*x*−*A*3))) + *A*4] (Kent *et al*. 1972, Matsumura *et al*. 1998b, Nagura *et al*. 2004) whereby *y* is the RSNA, *A*1 is the range over which the baroreflex curve operates, *A*2 is the sensitivity, *A*3 is the value of *x* at mid-point of the curve and *A*4 is the lowest point to which RSNA or HR could be driven. Average values of RSNA and HR were calculated for each 5-mmHg change in blood pressure using averaged parameters of the equation for all the rats per group (Kopp *et al*. 2008). To detect the differences between each of the parameters (A1-A4) in the first and second baroreflex curves, a two-tailed paired Student's *t*-test was utilized. Data in Figure 5 were analysed using one-way anova followed by a Bonferroni *post hoc* test and unpaired *t*-test for specific group comparisons (GraphPad Prism software, GraphPad Prism® 5.0 for Windows, San Diego, CA, USA). Data were analysed offline and presented as mean ± SEM, and *P* values of <5% were accepted as statistically significant.

## Results

### Baseline haemodynamic and RSNA values

The baseline values of MAP, HR and RSNA were similar before and after saline infusion in the time control group except for the significant reduction (*P* < 0.05) in baseline HR in the Saline/PD group (Table 1). Similarly, the I.C.V. infusion of PD, CGP and losartan resulted in no significant change in the baseline values of MAP, HR or RSNA except for the significant decrease (*P* < 0.05) in baseline HR values in the CGP/CGP+L-NAME group.

**Table I:** Mean arterial pressure, heart rate, and renal sympathetic nerve activity obtained before and following the first and the second I.C.V. infusions

			First I.C.V. infusion			Second I.C.V. infusion	
	Parameter		Before	After		Before	After
Saline/Saline	MAP (mmHg)	*Saline*	86 ± 3	86 ± 2	*Saline*	84 ± 1	85 ± 1
	HR (bpm)		316 ± 8	317 ± 8		311 ± 6	314 ± 4
	RSNA (*μ*V.s)		0.98 ± 0.36	1.04 ± 0.39		0.97 ± 0.35	0.99 ± 0.34
Saline/PD	MAP (mmHg)	*Saline*	80 ± 2	79 ± 2	*PD*	80 ± 3	90 ± 2
	HR (bpm)		330 ± 17	318 ± 16*		318 ± 18	320 ± 20
	RSNA (*μ*V.s)		0.66 ± 0.15	0.76 ± 0.21		0.62 ± 0.15	0.68 ± 0.18
PD/PD+L-NAME	MAP (mmHg)	*PD*	84 ± 4	83 ± 4	*PD+L-NAME*	86 ± 4	88 ± 4
	HR (bpm)		321 ± 9	324 ± 14		320 ± 23	326 ± 21
	RSNA (*μ*V.s)		0.67 ± 0.21	0.69 ± 0.22		0.66 ± 0.29	0.77 ± 0.32
Saline/L-NAME	MAP (mmHg)	*Saline*	80 ± 7	81 ± 7	*L-NAME*	80 ± 11	82 ± 10
	HR (bpm)		325 ± 18	319 ± 17		316 ± 23	315 ± 22
	RSNA (*μ*V.s)		0.96 ± 0.09	1.05 ± 0.12		1.25 ± 0.43	1.37 ± 0.48
L-NAME/L-NAME+PD	MAP (mmHg)	*L-NAME*	86 ± 5	86 ± 5	*L-NAME+PD*	89 ± 4	87 ± 3
	HR (bpm)		310 ± 8	305 ± 11		304 ± 15	302 ± 12
	RSNA (*μ*V.s)		1.02 ± 0.30	1.07 ± 0.33		0.91 ± 0.33	0.95 ± 0.33
Saline/CGP	MAP (mmHg)	*Saline*	84 ± 7	83 ± 7	*CGP*	88 ± 6	86 ± 6
	HR (bpm)		313 ± 16	308 ± 16		309 ± 18	306 ± 20
	RSNA (*μ*V.s)		0.65 ± 0.11	0.65 ± 0.11		0.60 ± 0.09	0.60 ± 0.09
CGP/CGP+L-NAME	MAP (mmHg)	*CGP*	80 ± 4	81 ± 5	*CGP+L-NAME*	82 ± 3	80 ± 3
	HR (bpm)		316 ± 9	307 ± 10*		319 ± 17	330 ± 20
	RSNA (*μ*V.s)		0.87 ± 0.19	1.02 ± 0.27		0.81 ± 0.28	0.83 ± 0.28
Saline/Losartan	MAP (mmHg)	*Saline*	87 ± 6	88 ± 6	*Losartan*	96 ± 3	94 ± 4
	HR (bpm)		329 ± 11	328 ± 13		317 ± 18	336 ± 14
	RSNA (*μ*V.s)		0.69 ± 0.25	0.72 ± 0.25		0.75 ± 0.29	0.81 ± 0.31
Losartan/losartan+L-NAME	MAP (mmHg)	*Losartan*	81 ± 6	81 ± 7	*Losartan+L-NAME*	81 ± 8	78 ± 9
	HR (bpm)		335 ± 20	338 ± 19		344 ± 25	346 ± 26
	RSNA (*μ*V.s)		0.70 ± 0.12	0.78 ± 0.16		0.75 ± 0.24	0.77 ± 0.25

MAP, mean arterial pressure; HR, heart rate; RSNA, renal sympathetic nerve activity; I.C.V., intracerebroventricular.

Values are expressed as means ± SEM.

* *P*< 0.05 after compared to before I.C.V. infusion.

### Baroreflex curves of RSNA and HR

**Figure 1:**
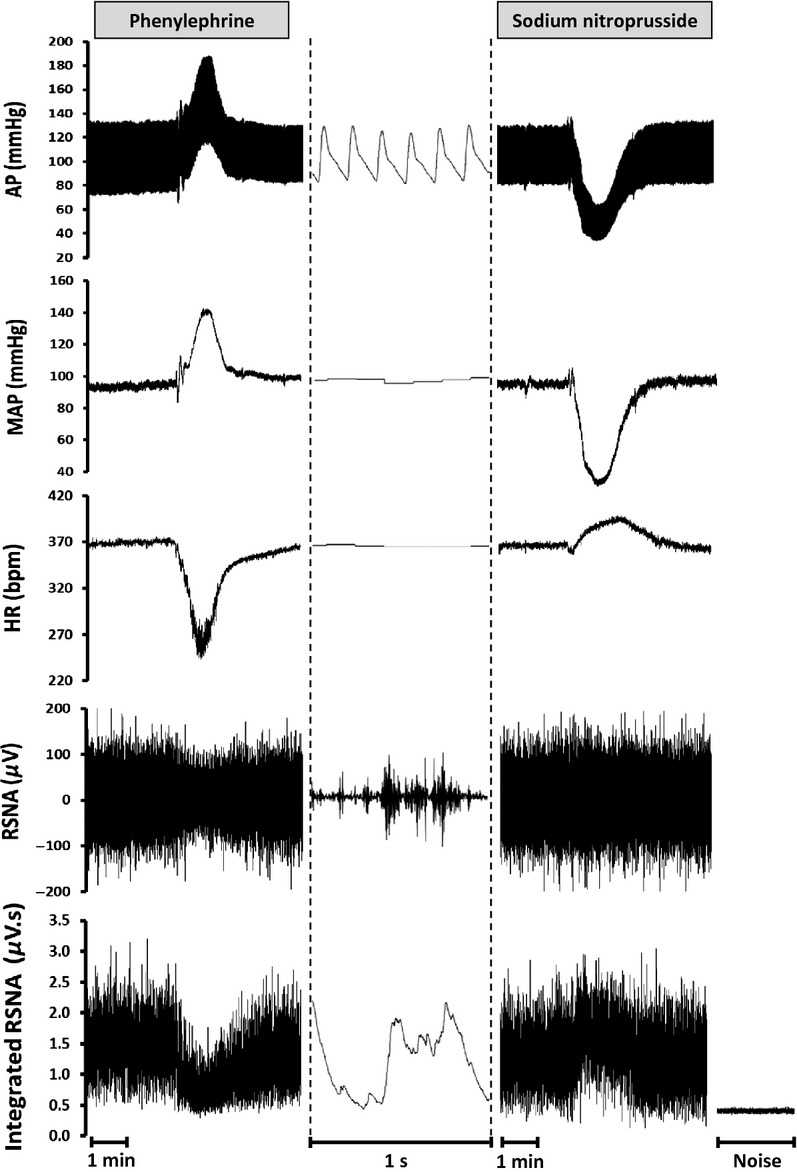
Representative recording from an individual rat of systemic arterial pressure (AP), mean arterial blood pressure (MAP), heart rate (HR), integrated renal sympathetic nerve activity (RSNA) and row RSNA signal. Ramp changes in AP were established by bolus intravenous infusions of phenylephrine (50 *μ*g kg^−1^) to increase AP and sodium nitroprusside (50 *μ*g kg^−1^) to decrease AP. Data are presented at two different recording speeds.

Figure 1 illustrates the raw and integrated RSNA, and HR recordings at baseline and when ramp changes in arterial pressure were triggered by PE and SNP infusion in one time control rat. PE induced an increase in MAP by almost 50 mmHg which was accompanied by a corresponding decrease in RSNA and HR. On the other hand, SNP produced a marked drop in MAP by almost 60 mmHg which triggered a parallel increase in RSNA and HR.

**Figure 2:**
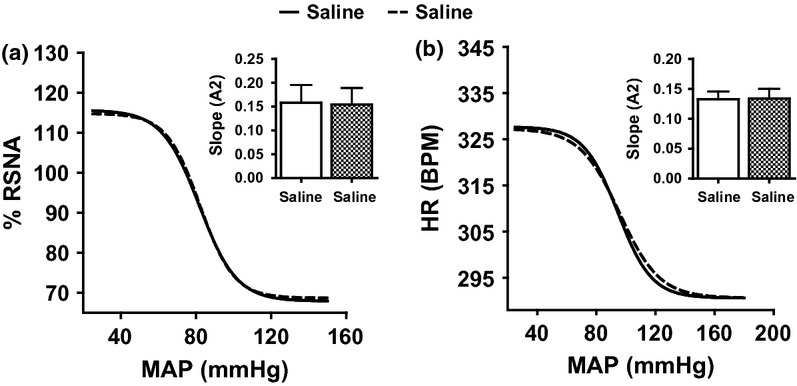
The baroreflex curve for renal sympathetic nerve activity (a) and heart rate (b) following ramp changes in mean arterial blood pressure in the time control group whereby a first I.C.V. saline infusion was followed by a second I.C.V. saline infusion. RSNA, renal sympathetic nerve activity; MAP, mean arterial blood pressure; and HR, heart rate.

Figures 5 contain the baroreflex curves for the relationship between RSNA or HR and MAP following I.C.V. administration of saline during the control phase and then following I.C.V. L-NAME, CGP, PD and losartan. In the time control group (saline/saline), the sensitivity (*A*2) of RSNA (Fig. 2a) and HR (Fig. 2b) baroreflex curves following I.C.V. saline in the first phase was comparable to those recorded following I.C.V. saline administration in the second phase.

**Figure 3:**
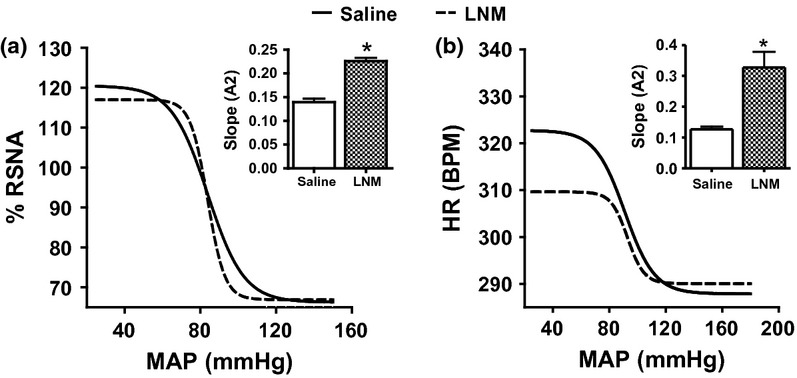
The baroreflex curve for renal sympathetic nerve activity (a) and heart rate (b) following ramp changes in mean arterial blood pressure in I.C.V. saline infused rats followed by L-NAME. *P < 0.05, baroreflex sensitivity of the second phase compared with the first phase. LNM, L-NAME; RSNA, renal sympathetic nerve activity; MAP, mean arterial blood pressure; and HR, heart rate.

**Figure 4:**
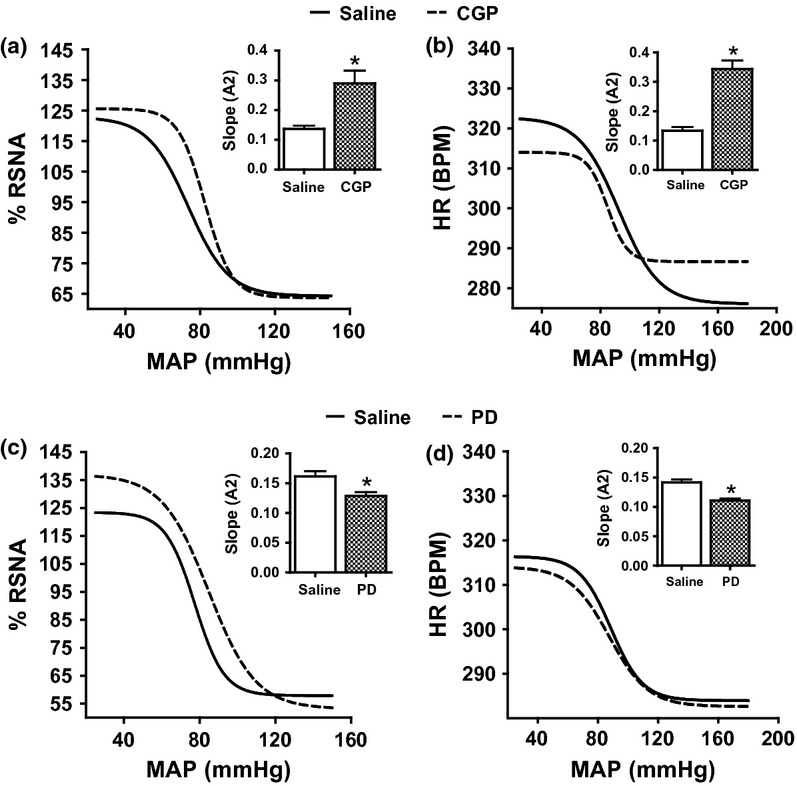
The baroreflex curve for renal sympathetic nerve activity and heart rate following ramp changes in mean arterial blood pressure in I.C.V. saline infused rats followed by CGP (a, b) or PD (c, d). *P < 0.05, baroreflex sensitivity of the second phase compared with the first phase. PD, PD123319; RSNA, renal sympathetic nerve activity; MAP, mean arterial blood pressure; and HR, heart rate.

**Figure 5:**
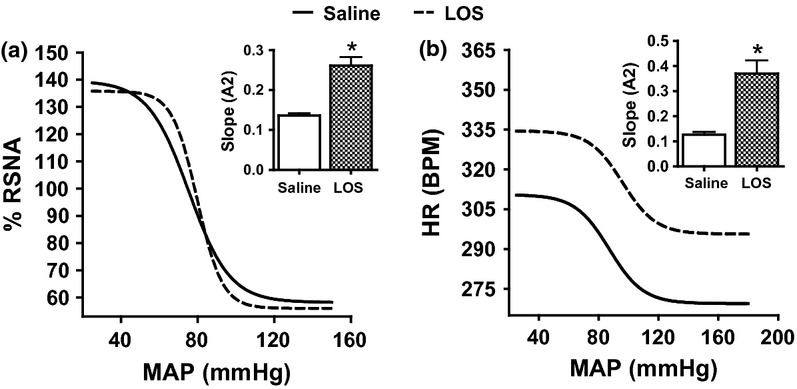
The baroreflex curve for renal sympathetic nerve activity (a) and heart rate (b) following ramp changes in mean arterial blood pressure in I.C.V. saline infused rats followed by losartan. *P < 0.05, baroreflex sensitivity of the second phase compared with the first phase. LOS, losartan; RSNA, renal sympathetic nerve activity; MAP, mean arterial blood pressure; and HR, heart rate.

Figure 8 summarizes the percentage change in the sensitivity (*A*2) of the baroreflex curve of RSNA or HR in all experimental groups. The second saline phase of saline/saline group was utilized as a baseline level, and the percentage increase or decrease in the sensitivity (*A*2) of the second baroreflex curve from this baseline in each group was calculated.

### Role of NO

To investigate the impact of central NO on the baroreflex control mechanism of RSNA and HR, baroreflex curves were generated before and then after L-NAME administration. Infusion of L-NAME I.C.V. resulted in a significant increase of 50–60% in the RSNA (Figs 3a and 8a) and approx. 150–160% in HR (Figs 3b and 8b) baroreflex curve sensitivities (A2) compared with those when saline was given I.C.V. respectively. However, the mid-point (*A*3) and the lower plateau (*A*4) of these curves remained unchanged except for the range (*A*1) of HR baroreflex which decreased significantly by some 43% (*P* < 0.05) following NOS blockade with L-NAME.

### Role of AT2 receptors

The role of central AT2 receptors on the baroreflex mechanism of RSNA and HR was investigated by generation of baroreflex curves before and following the I.C.V. administration of CGP or PD. Infusion of CGP I.C.V. resulted in a significant increase of 90–110 and 150% (all *P* < 0.05) in the sensitivity (*A*2) of the RSNA (Figs 4a and 8a) and HR (Figs 4b and 8b) baroreflex curves, respectively, compared with those obtained when saline was administered I.C.V. Conversely, I.C.V. administration of PD decreased the RSNA (Figs 4c and 8a) and HR (Figs 4d and 8b) baroreflex sensitivities, A2, (all *P* < 0.05) by some 20%. The administration of either CGP or PD resulted in no significant change in the range (*A*1), mid-point (*A*3) or lower plateau (*A*4) of the RSNA (Fig. 4a, c) or HR (Fig. 4b, d) curves compared with saline I.C.V. administration.

### Role of AT1 receptors

The impact of central AT1 receptor blockade on the baroreflex control of RSNA and HR was examined by studying the baroreflex curves before and following the I.C.V. administration of AT1 antagonist, losartan. The administration of losartan I.C.V. significantly increased the sensitivity (*A*2) of the RSNA baroreflex by about 80–90% (Fig. 5a, Fig. 8a) and for HR baroreflex by 150–190% (Figs 5b and 8b) (both *P* < 0.05), respectively, compared with saline I.C.V. administration without change to any other of the parameters.

### The effect of NOS blockade on central AT2 receptors

**Figure 6:**
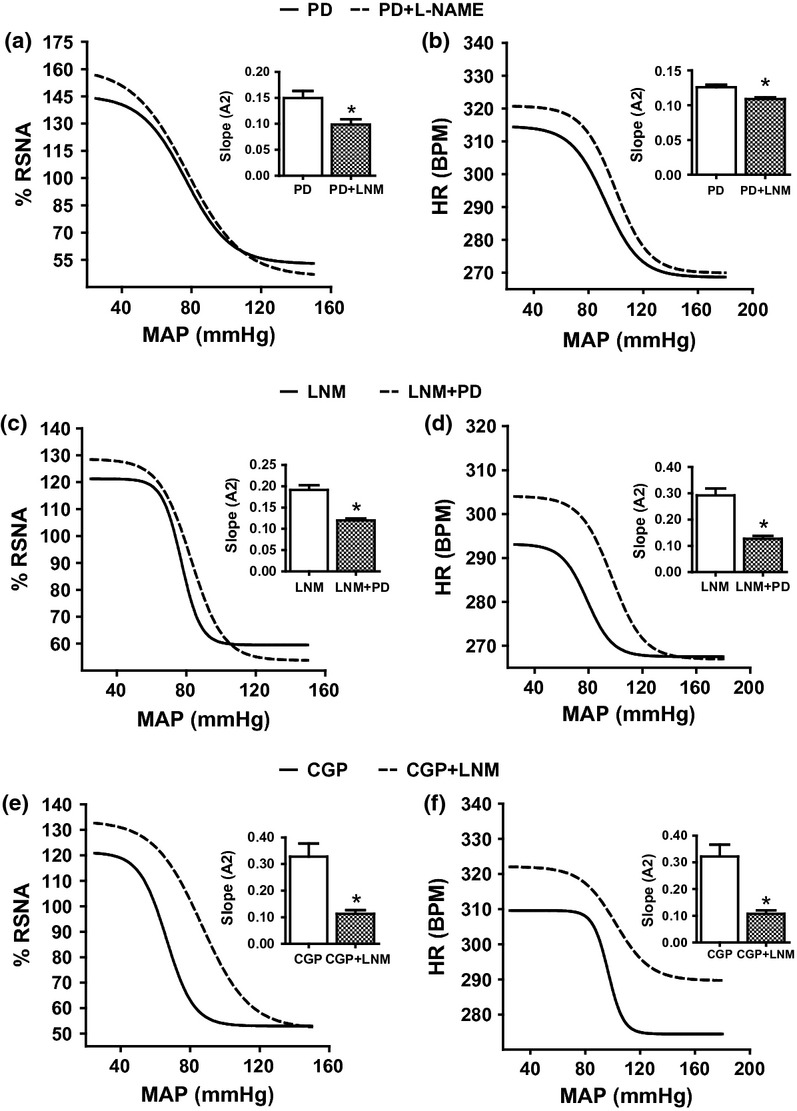
The baroreflex curve for renal sympathetic nerve activity and heart rate following ramp changes in mean arterial blood pressure in PD/PD+L-NAME (a, b), L-NAME/L-NAME+PD (c, d) and CGP/CGP+L-NAME (e, f). *P < 0.05, baroreflex sensitivity of the second phase compared with the first phase. LNM, L-NAME; PD, PD123319; RSNA, renal sympathetic nerve activity; MAP, mean arterial blood pressure; and HR, heart rate.

L-NAME was co-infused with CGP or PD to determine the effect of central NOS blockade on AT2 receptor-mediated impact on the baroreflex mechanism. The administration of L-NAME in combination with CGP I.C.V. in CGP/CGP+L-NAME group decreased the sensitivity (*A*2) of the baroreflex curve significantly to values not different from saline control values for RSNA (Figs 6a and 8a) and HR (Figs 6b and 8b) (both *P* < 0.05). The other parameters of the baroreflex curve remained the same following combined administration of L-NAME plus CGP except for the effect of L-NAME on the mid-point (*A*3) of the RSNA baroreflex gain curve which increased significantly by some 15% (from 75.6 ± 5.2 to 84.7 ± 5.9, *P* < 0.05) (Fig. 6a).

The combined administration of L-NAME with PD I.C.V. in PD/PD+L-NAME or L-NAME/PD+L-NAME group produced a significantly lower (*P* < 0.05) sensitivity (*A*2) in both groups (Figs 6c,e and 8a) for RSNA some 13% and 57% for HR (Figs 6d,f and 8b) compared with I.C.V. infusion of PD or L-NAME which were values very similar to those obtained when saline alone was infused. There was no effect on the range (*A*1), the mid-point (*A*3) or the lower plateau (*A*4) of the curve except that the mid-point of HR baroreflex curve (*A*3) increased by almost 25% (from 80.5 ± 4.9 to 100.6 ± 5.9, *P* < 0.05) following L-NAME I.C.V. administration in the L-NAME/L-NAME+PD group.

### The effect of central NOS and AT1 receptors blockade

**Figure 7:**
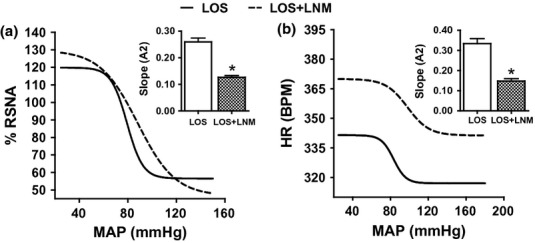
The baroreflex curve for renal sympathetic nerve activity (a) and heart rate (b) following ramp changes in mean arterial blood pressure in losartan/losartan+L-NAME. *P < 0.05, baroreflex sensitivity of the second phase compared with the first phase. LOS, losartan; LNM, L-NAME; RSNA, renal sympathetic nerve activity; MAP, mean arterial blood pressure; and HR, heart rate.

**Figure 8:**
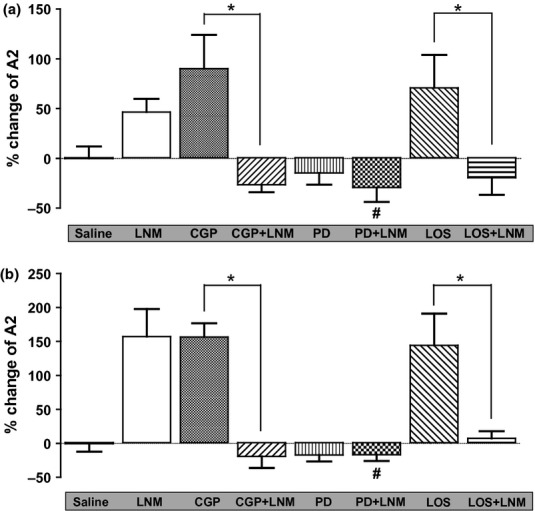
The percentage change in baroreflex sensitivity (A2) of RSNA (a) and HR (b) baroreflex gain curves from control (saline) in the presence and absence of L-NAME. The data are for the percentage difference of the slope values (A2) of the second baroreflex curves in all groups with reference to the slope of second saline phase in saline/saline group as a baseline value. *P < 0.05, with L-NAME compared to without L-NAME; #*P* < 0.05, PD+L-NAME compared with L-NAME. LNM, L-NAME; PD, PD123319; and LOS, losartan.

To investigate the effects of combined blockade of NOS and AT1 receptors on the baroreflex mechanism, L-NAME was co-infused with losartan and the baroreflex curves for RSNA or HR was generated. The sensitivity (*A*2) of the baroreflex curves for RSNA (Figs 7a, and 8a) and HR (Figs 7b and 8b) was significantly blunted (all *P* < 0.05), respectively, following the I.C.V. infusion of L-NAME in combination with losartan compared with losartan I.C.V. infusion. In addition, L-NAME administration increased the mid-point (*A*3) of the RSNA or HR baroreflex curves by some 11% and 17% respectively (all *P* < 0.05). There was no significant change in any other parameter of the baroreflex curve following combined administration of L-NAME plus losartan (Fig. 7).

## Discussion

The aim of the present study was to investigate whether there was a contribution of NO to the high-pressure baroreceptor reflex regulation of RSNA and HR mediated through central AT2 receptors. The approach taken was to generate baroreflex curves to determine the relationship between MAP and RSNA or HR before and after AT2 receptor stimulation or blockade with NOS active or inhibited with L-NAME. The contribution of AT1 receptors was similarly examined. Several novel findings were revealed; firstly, under basal conditions, both the renin-angiotensin and NO systems contributed significantly to the high-pressure baroreceptor control of RSNA and HR; secondly, exogenous stimulation of AT2 receptors by CGP increased the sensitivity of the baroreceptor reflex which was dependent, in large part, on an intact NO system, whereas blockade of AT2 receptors with PD caused a small decrease in sensitivity which was marginally increased by subsequent blockade of the NO system; thirdly, a similar pattern of responses was observed when the counterbalancing AT1 receptors were blocked with losartan. In this regard, there was a significant increase in the sensitivity of RSNA or HR baroreflexes following AT1 receptor blockade, but when central NOS was blocked with L-NAME, this increase in baroreflex sensitivity was reversed.

The rats in the current study were subjected to two I.C.V. injections of either vehicle, agonist or antagonist, and during each of the two I.C.V. administrations, baroreflex curves for RSNA and HR were generated. Previous studies have indicated that I.C.V. administration of substances could have different sites of action and therefore different effects on the baroreflex mechanism (Ferguson & Washburn 1998, Chen & Toney 2003, Fletcher *et al*. 2006). For example, Ang II was suggested to blunt the baroreflex mechanism due to its effect on a subpopulation of NTS neurones (Kasparov & Paton 1999). However, in another study, the effect of Ang II on RVLM neurones was described as sympatho-excitatory (Head 1996). In a similar fashion, NO within the RVLM was found to exert a GABA-mediated tonic inhibitory effect on RSNA (Zhang & Patel 1998). Furthermore, the NO effect on PVN was such that it produced a decrease in RSNA, MAP and HR in rats (Zhang *et al*. 1997). However, in the NTS neurones, NO was shown to have a facilitatory role on the baroreflex mechanism in rats (Dias *et al*. 2005). The time control group showed similar baseline haemodynamic and RSNA parameters, and baroreflex curves in the first and second phases of the protocol, indicating that there was no effect of time on either the baseline RSNA, MAP or HR values or the baroreceptor reflex regulation of these variables. It is important to emphasize that these studies were performed in an anaesthetized preparation and that the changes in RSNA and HR in response to the vasopressor and vasodepressor compounds were relatively blunted compared with those obtained in conscious preparations (M.H. Abdulla & E.J. Johns, unpublished observations).

The baseline values of RSNA, MAP and HR in all groups did not change following the central administration of PD, CGP or losartan which agrees with previous reports from this laboratory and from others (Bunting & Widdop 1995, Oliveira *et al*. 1996, Abdulla & Johns 2013). In the present study, the I.C.V. administration of L-NAME into the lateral ventricle caused no significant change in RSNA, MAP or HR which was similar to that previously reported in the mouse using comparable doses of L-NAME I.C.V. (Moore *et al*. 1991) although it has been reported that central injection of the NOS inhibitor, L-NAME resulted in a higher level of RSNA (Togashi *et al*. 1992). In terms of the AT1 receptor blocker, losartan had no significant effect on baseline levels of MAP, HR or RSNA which was consistent with previous studies in rats (Huang *et al*. 2006) and rabbits (Badoer *et al*. 2000) given a dose of losartan close to that used in the present study.

An important novel finding from the present study was that inhibition of NO production in the central nervous system with L-NAME enhanced baroreflex sensitivity to RSNA as well as to HR. This would suggest that NO normally exerts a tonic inhibitory action at the level of baroreflex regulation of both RSNA and HR. These findings support those of other groups in that NO was found to attenuate the baroreceptor-mediated reflex regulation of RSNA and HR in conscious rabbits (Matsumura *et al*. 1998a). Therefore, this study as well as previous reports indicate that central NO possesses an important cardiovascular regulatory role which may reflect its action as a neurotransmitter or neuromodulator (Togashi *et al*. 1992).

Following the I.C.V. administration of PD to block AT2 receptors, the baroreflex control of RSNA and HR was suppressed. This finding would indicate that under basal conditions, AT2 receptor activation by angiotensin II contributed to the baroreflex regulation of RSNA and HR. Indeed, there are reports demonstrating that AT2 receptors are expressed in brain areas related to sympathetic control such as rostral ventrolateral medulla (RVLM), NTS and the subfornical organ (Lenkei *et al*. 1997, Roulston *et al*. 2003, Gao *et al*. 2008). In addition, AT2 receptor activation produced a sympatho-inhibition which was postulated to be related to central NO production (Gao *et al*. 2008, Gao & Zucker 2010). Although these studies defined the effect on basal levels of renal sympathetic nerve activity, what was not tested was their action on baroreflex sensitivity.

A second novel and important observation was that I.C.V. administration of CGP in the current study increased baroreflex sensitivity for RSNA and HR compared with the vehicle. These findings are at variance with previous studies which showed a tendency for CGP I.C.V. to decrease the slope of the HR baroreflex sensitivity in normotensive rats (Oliveira *et al*. 1996). One reason for this difference is possibly due to the different sites at which this agonist may be acting along the baroreflex pathway as emphasized above. However, the microinjection of CGP into the RVLM produced a sympatho-inhibitory response in rats using a dose comparable to that utilized in the present study (Gao *et al*. 2008). This sympatho-inhibitory response was also accompanied by a decrease in MAP, HR and RSNA. In addition, data from mice with deleted AT2 receptor gene demonstrated a depressor response to central AT2 receptor stimulation buffering an AT1 receptor-mediated pressor response (Gross *et al*. 2002). The I.C.V. administration of CGP in the current study as well as in that of Oliveira *et al*. (1996) had no effect on baseline MAP. However, in these studies, either the AT2 receptor-mediated alterations in baseline levels of sympathetic outflow were measured or an indirect measure of baroreflex sensitivity based on heart rate and blood pressure changes was utilized but baroreflex control of sympathetic nerve activity was not evaluated. The increase in baroreflex sensitivity due to CGP was significantly reversed following the co-infusion of L-NAME and the response was shifted to a higher blood pressure level. This would be compatible with the view that exogenous activation of central AT2 receptors exerts its effect, to a large degree, through a pathway involving NO production.

It was also evident that losartan increased the RSNA and HR baroreflex sensitivity. This finding is similar to previous reports from this laboratory and others who found that injection of losartan either I.C.V. or onto the NTS enhanced the sensitivity of the high-pressure baroreflex control of RSNA or HR in conscious and anaesthetized rats (Oliveira *et al*. 1996, Huang *et al*. 2006, Wang *et al*. 2007). Moreover, an enhanced sympatho-inhibitory response to volume expansion was reported following I.C.V. administration of losartan in rats (Dibona *et al*. 1998, Abdulla & Johns 2013). These findings may indicate an important AT2 receptor-mediated role for endogenous angiotensin II to enhance the baroreflex regulation of RSNA and HR. This suggestion is based on the notion that AT2 receptor function becomes apparent only when the AT1 receptors are blocked (Israel *et al*. 2000). The AT2 receptor-mediated effect on the baroreflex regulatory mechanisms is dependent on a functional NO system within the brain. This suggestion is supported by the present observation that L-NAME co-administration with losartan reversed the effect of losartan on the RSNA and HR baroreceptor reflex. The magnitude of the decrease in baroreflex sensitivity in response to co-infusion of losartan with L-NAME was comparable to the decrease in baroreflex sensitivity following CGP infusion in the presence of NOS blockade. However, this relationship is only for the high-pressure baroreflex mechanism but not the sympatho-inhibitory response mediated by the low-pressure cardiopulmonary baroreceptors as recently shown using a similar manipulation of central AT1 and AT2 receptors (Abdulla & Johns 2013).

In conclusion, the findings from the current study suggest that a functional NO system within the brain has an important regulatory role in the normal baroreflex regulation of RSNA and HR which is initiated by the activation of central AT2 receptors. That is, when AT2 receptors were exogenously activated or when the counterbalancing actions of AT1 receptors were blocked, an augmented sensitivity of the high-pressure baroreceptors became evident. Part of this response is dependent on AT2-mediated NO production as when L-NAME was given the AT2 receptor-induced increase in the baroreflex sensitivity was blunted. The outcome of this study provides a clearer insight as to the significance of AT2 receptors in the brain in regulating sympathetic outflow to the kidneys. This relationship may become important when attempting to understand the impaired baroreflex mechanisms existing in renal failure and hypertension.

## Conflict of interest

There is no conflict of interest by the authors.

This work was funded by the Wellcome Trust.

## Author contributions

The authors have equally contributed to the design of the experiments, data collection, interpretation and manuscript revision.
